# Mitochondrial-encoded membrane protein transcripts are pyrimidine-rich while soluble protein transcripts and ribosomal RNA are purine-rich

**DOI:** 10.1186/1471-2164-6-136

**Published:** 2005-09-26

**Authors:** Patrick C Bradshaw, Anand Rathi, David C Samuels

**Affiliations:** 1 Virginia Bioinformatics Institute, Virginia Polytechnic Institute and State University, Blacksburg, VA 24061, USA

## Abstract

**Background:**

Eukaryotic organisms contain mitochondria, organelles capable of producing large amounts of ATP by oxidative phosphorylation. Each cell contains many mitochondria with many copies of mitochondrial DNA in each organelle. The mitochondrial DNA encodes a small but functionally critical portion of the oxidative phosphorylation machinery, a few other species-specific proteins, and the rRNA and tRNA used for the translation of these transcripts. Because the microenvironment of the mitochondrion is unique, mitochondrial genes may be subject to different selectional pressures than those affecting nuclear genes.

**Results:**

From an analysis of the mitochondrial genomes of a wide range of eukaryotic species we show that there are three simple rules for the pyrimidine and purine abundances in mitochondrial DNA transcripts. Mitochondrial membrane protein transcripts are pyrimidine rich, rRNA transcripts are purine-rich and the soluble protein transcripts are purine-rich. The transitions between pyrimidine and purine-rich regions of the genomes are rapid and are easily visible on a pyrimidine-purine walk graph. These rules are followed, with few exceptions, independent of which strand encodes the gene. Despite the robustness of these rules across a diverse set of species, the magnitude of the differences between the pyrimidine and purine content is fairly small. Typically, the mitochondrial membrane protein transcripts have a pyrimidine richness of 56%, the rRNA transcripts are 55% purine, and the soluble protein transcripts are only 53% purine.

**Conclusion:**

The pyrimidine richness of mitochondrial-encoded membrane protein transcripts is partly driven by U nucleotides in the second codon position in all species, which yields hydrophobic amino acids. The purine-richness of soluble protein transcripts is mainly driven by A nucleotides in the first codon position. The purine-richness of rRNA is also due to an abundance of A nucleotides. Possible mechanisms as to how these trends are maintained in mtDNA genomes of such diverse ancestry, size and variability of A-T richness are discussed.

## Background

Mitochondria are the descendents of an early bacterium that developed a symbiotic relationship with another cell approximately 1.5 billion years ago [1]. Although the mitochondria still contain DNA, the mitochondrial genome has greatly simplified over its long history of symbiosis. Naturally, this simplification in the mitochondrial genome has taken different routes as life diverged into different kingdoms. Vertebrate mitochondrial genomes are among the most compact, gene-rich genomes, while some plant mitochondria have evolved to have a low percentage of coding region similar to that of nuclear DNA [2]. Features of the mitochondrial genomes that have persisted through the divergent evolution of eukaryotic life are likely to be due to fundamental limitations on the variation of that genome. In this paper we discuss three such features that are preserved across eukaryotic species.

Because of the relatively small size of mitochondrial DNA, it is ideally suited for analysis by *n*-dimensional DNA walks. One dimensional pyrimidine-purine walks were first used to find long-range correlations in nucleotide sequences [3]. Recently multi-fractal walks of mitochondrial DNA were used to find a nonlinear organization in the mitochondrial genome [4]. Combining this information with pyrimidine-purine walks and walks of G-C versus A-T content [5] gives a better understanding of the nucleotide organization of the genome.

Using these techniques we demonstrate certain features of mtDNA sequences which have been preserved by evolution. Greater understanding of the evolutionary selection pressures on mtDNA will allow the construction of more accurate phylogenetic trees based upon mtDNA gene sequences [6,7] as well as a better grasp of the root causes of mitochondrial DNA mutations responsible for many human diseases.

## Results

**Figure 1:**
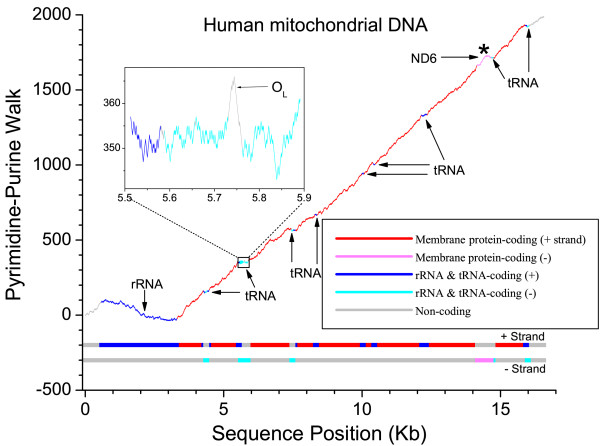
**A pyrimidine-purine walk of human (+ strand) mtDNA**. Genes having mRNA synonymous with the (+) strand or (-) strand are indicated by color and also shown on the strand bars below the graph. An inset of a tRNA-containing section of the graph around the origin of light strand replication (O_L_) is shown.

A pyrimidine (C and T) – purine (A and G) walk of the (+) strand of human mtDNA (commonly called the "light" strand in vertebrates) is shown in Figure 1. For each pyrimidine in the sequence a step up is taken and for each purine a step down is taken. In vertebrates, all mitochondrial genes except ND6 and many tRNAs are encoded on the heavy strand. Therefore the mRNA species are predominantly (+) light strand synonymous. The first 3-kilobase section of the human mtDNA encodes two ribosomal RNAs and the pyrimidine-purine walk slopes downward in this region, indicating that the ribosomal RNAs are slightly purine-rich. The remainder of the genome predominately encodes mitochondrial oxidative phosphorylation proteins with small tRNAs interspersed between them. Each mitochondrial protein transcript (except ND6) is pyrimidine-rich giving the remainder of the graph an upward slope. Within this overall rise, small almost flat sections where tRNA genes are located can be seen between the protein coding regions. A particularly large group of these tRNAs is contained in the section of mtDNA around the origin of light strand replication (O_L_) (shown as an inset to Figure 1). The O_L _is dramatically clear in this plot as a large run of pyrimidines on one side of the O_L _followed by a long run of purines on the other side. This section is thought to form a stem-loop structure as an initiation event for light strand DNA synthesis [8,9]. However, we should note that a stem-loop structure does not require a dramatic separation of pyrimidines and purines as is seen here. The ND6 gene has a slightly upward slope in the walk indicating that the human ND6 mRNA is slightly purine-rich in contrast to the other protein-coding mRNAs encoded on the opposite strand.

**Figure 2:**
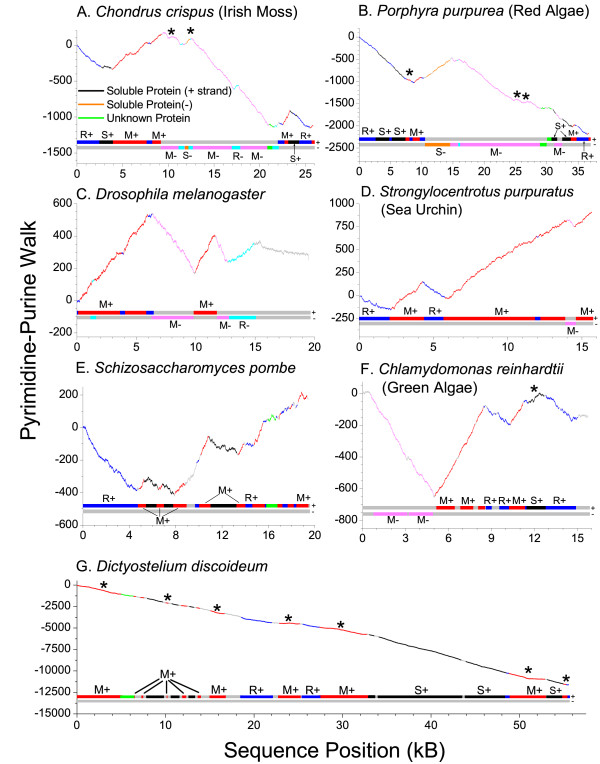
**Pyrimidine-purine walks of mitochondrial genome (+) strands of selected species**. M, S, and R indicate membrane protein-coding, soluble protein-coding, and RNA-coding segments, respectively. Single tRNA genes are not shown due to their small size, but stretches of 2 or more consecutive tRNAs on the same strand are shown. The coloring scheme for colors not shown in the legend follows that of Figure 1.

Clear pyrimidine and purine rich genome segments can also be seen in the mtDNA from other eukaryotic species. The pyrimidine-purine walks of the mitochondrial genome of seven diverse species are shown in Figure 2. In these species many of the genes, the gene order, and the gene distribution over the two DNA strands are different. Figure 2A shows a pyrimidine-purine walk of the mitochondrial genome of the red algae *Chondrus crispus *(Irish Moss). The genes are color-coded based upon whether they encode membrane proteins, soluble proteins, or rRNA and upon the strand in which they are encoded. Figure 2B shows a mitochondrial genome walk of the red algae, *Porphyra purpurea*. The similar gene order in these two red algae species (Figures 2A and 2B) gives the walks a similar overall shape. Metazoan mitochondrial genomes such as those shown from Drosophila (Figure 2C) and sea urchin (Figure 2D) are pyrimidine rich overall (+ strands), especially in the sea urchin where all but one of the genes are encoded on the same strand. Plant, fungal, and protist genomes that are gene-poor, which encode oxidative phosphorylation proteins on both strands, and also encode soluble proteins are generally purine-rich. Small genomes (<25 kB), such as that of *Schizosaccharomyces pombe *(fission yeast) (Figure 2E), are pyrimidine-rich because they are gene-rich and encoded entirely on one strand.

No matter which strand encodes the genes, or whether the entire mitochondrial genome is pyrimidine or purine-rich, there are highly conserved features in these walks. Places in the genome where the DNA walk went down were locations where rRNA or soluble proteins were encoded on the (-) (heavy) strand or where membrane proteins were encoded on the (+) (light) strand. Locations of membrane proteins on the (-) strand or rRNA or soluble proteins on the (+) strand were associated with an upward slope in the pyrimidine-purine walk. Exceptions to these rules are indicated on the figure with an asterisk. The most notable exception that we found was the mtDNA from the slime mold *Dictyostelium discoideum *(Figure 2G), which has a very strand-asymmetric genome, being very purine-rich on the (+) strand (60%). Unlike other species, in *Dictyostelium *almost all oxidative-phosphorylation membrane-complex transcripts on the (+) strand were purine-rich, just like the rest of its genome. To place the extreme purine richness of *Dictyostelium *in context, the human mtDNA genome (+) strand is 44 % purine while that of the plant *Arabidopsis thaliana *is 50 % purine. The purine abundance of the mitochondrial genome of other species is listed in additional file 1: Table S1. Slime molds contain the most purine-rich (+) strand of any of the 23 organisms we examined, while mammals, birds, and a green algae species, *Pedinomonas minor*, contain the least amount of purine (44%).

We analyzed the pyrimidine and purine content of mitochondrial transcripts from many diverse eukaryotic species (Table 1). Unlike vertebrate mtDNA that lacks genes for soluble proteins, plant, fungi, and protist mtDNA encode genes for many ribosomal proteins and a few other soluble proteins. From this data we defined the following three rules that apply to the pyrimidine-purine richness of mitochondrial transcripts.

**Table 1:** The number of genes that obey the rules of mitochondrial pyrimidine-purine base composition. All oxidative phosphorylation complex protein genes were included as membrane protein genes. Soluble mitochondrial protein genes included those of ribosomal proteins, maturases and endonucleases from intronic ORFs, and polymerase-like proteins. Unknown ORFs, hypothetical proteins, and proteins of unknown localization were excluded from the analysis. Transcripts that do not follow Rule #1 include almost all *Dictyostelium *transcripts, *Chondrus crispus SDH2*, *Porphyra purpurea SDH2*, *COX2*, and *ymf39*, *Marchantia polymorpha NAD7 *and *ATPa*, and *Arabidopsis NAD7*, *NAD9*, and *ATP1*. The *Chlamydomonas reinhardtii rtl *transcript breaks Rule #3.

	**Organism Name**		**Rule 1**	**Rule 2**	**Rule 3**	**Hypothetical Proteins or Unknown Localization**
Genbank Accession	**Latin**	**Common**	Holds **(Fails)**	**Holds (Fails)**	**Holds (Fails)**	

[NC_001807]	*Homo sapiens*	Human	12 (1)	2	-	-
[NC_005089]	*Mus musculus*	Mouse	12 (1)	2	-	-
[NC_001913]	*Oryctolagus cuniculus*	Rabbit	12 (1)	2	-	-
[NC_002008]	*Canis familiaris*	Dog	13	2	-	-
[NC_000845]	*Sus scrofa*	Pig	13	2	-	-
[NC_001323]	*Gallus gallus*	Chicken	13	2	-	-
[NC_002784]	*Dromaius novaehollandiae*	Emu	13	2	-	-
[NC_001573]	*Xenopus laevis*	Frog	13	2	-	-
[NC_002333]	*Danio rerio*	Zebrafish	13	2	-	-
[NC_001709]	*Drosophila melanogaster*	Fruit fly	13	2	-	-
[NC_002074]	*Rhipicephalus sanguineus*	Brown dog tick	13	2	-	-
[NC_003344]	*Thyropygus sp. DVL-2001*	Giant millipede	13	2	-	-
[NC_001453]	*Strongylocentrot. purpuratus*	Sea urchin	13	2	-	-
[NC_000933]	*Metridium senile*	Brown sea anemone	13	2	1	-
[NC_001328]	*Caenorhabditis elegans*	Soil nematode	12	1 (1)	-	-
[NC_001224]	*Saccharomyces cerevisiae*	Baker's yeast	7	3	11	1
[NC_001326]	*Schizosaccharomyces pombe*	Fission yeast	6	2	-	4
[NC_000895]	*Dictyostelium discoideum*	Slime mold	2(13)	2	14	11
[NC_001677]	*Chondrus crispus*	Irish moss/Red algae	18(1)	2 (1)	5	1
[NC_002007]	*Porphyra purpurea*	Seaweed/Red algae	16(3)	2	6	6
[NC_001638]	*Chlamydomonas reinhardtii*	Green algae	7	11(3)	0(1)	-
[NC_000892]	*Pedinomonas minor*	Green algae	11	3	-	-
[NC_001660]	*Marchantia polymorpha*	Liverwort	13(2)	3	17	43
[NC_001284]	*Arabidopsis thaliana*	Thale cress	19(3)	2 (1)	8	87

Rule 1) Oxidative phosphorylation complex and other membrane protein transcripts are pyrimidine-rich.

Rule 2) Ribosomal RNA is purine-rich.

Rule 3) Soluble protein transcripts are purine-rich.

Table 1 lists the number of genes in each species that follow each rule, along with the number that fail. There were few exceptions to these rules. In some mammals (though not all) the ND6 transcript does not follow rule #1. The main exception for rule #2 is the large ribosomal RNA subunit in *C. elegans*, which has almost equal numbers of purines and pyrimidines. In other non-animal species the short 5S rRNA sometimes contains more pyrimidines than purines.

**Figure 3:**
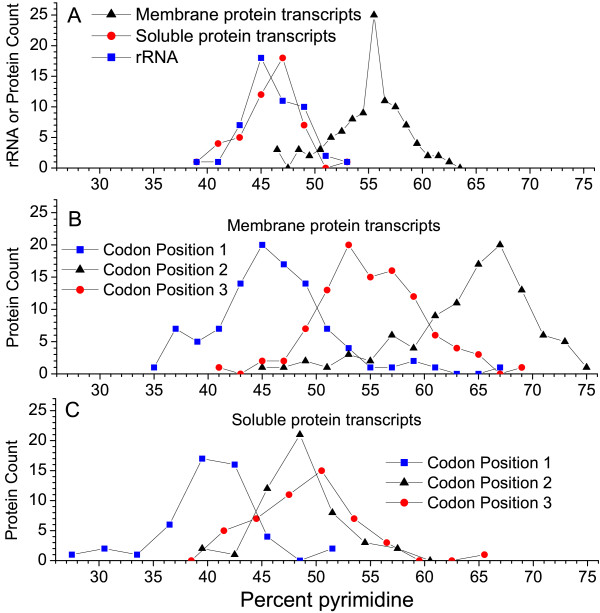
**Pyrimidine abundance in mitochondrial-encoded rRNA and codon positions in membrane and soluble protein transcripts**. (A) Complete transcripts (B) Codon positions in membrane protein transcripts (C) Codon positions in soluble protein transcipts. Mitochondrial genes from *Arabidopsis thaliana*, *Marchantia polymorpha*, *Chlamydomonas reinhardtii*, *Chondrus crispus*, *Porphyra purpurea*, *Saccharomyces cerevisiae*, and *Metridium senile *were analyzed. Unknown ORFS and hypothetical genes were excluded.

We examined the mtDNA from 8 species that encode genes for both soluble and membrane proteins. In Figure 3A we plot the percent pyrimidine in the transcripts versus the frequency at which transcripts of that type (soluble proteins, membrane proteins, or rRNA) occur in the 8 species. The membrane protein transcripts had a distinctive distribution with a peak at around 56 % pyrimidine. The ribosomal RNA and soluble protein transcripts had overlapping distributions with peaks near 45 % and 47 % pyrimidine respectively. These data explain the signals obtained in the pyrimidine-purine walks. It also gives an explanation as to why the soluble protein transcript walks are more variable than the membrane protein transcript walks, since the purine-rich signal is weaker in soluble protein transcripts than is the pyrimidine-rich signal in the membrane protein transcripts. The relative pyrimidine percentage at each codon position in membrane and soluble protein transcripts is shown in Figures 3B and 3C will be discussed later.

**Figure 4:**
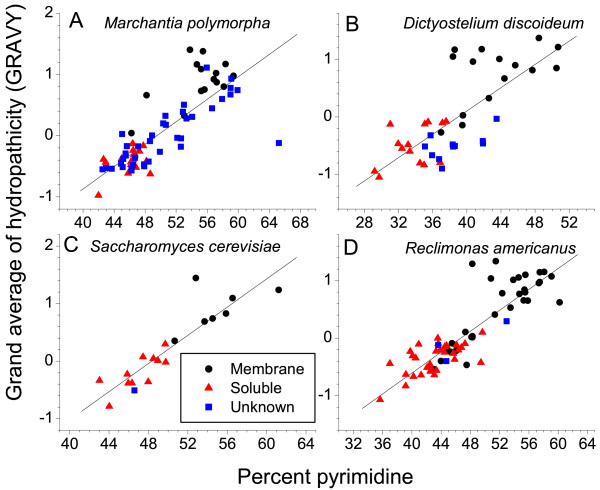
**The correlation between the hydrophobicity of a mitochondrial transcript and its pyrimidine content**. GRAVY scores were calculated using the ExPASy ProtParam website. Linear fit *P*-values were less than 0.001 for all panels. Linear fit *R*-values were (A) 0.82 (B) 0.75 (C) 0.90, and (D) 0.88. The numbers of membrane, soluble, and unknown protein-coding genes for the species in panels A-C are found in Table 1.

To clearly illustrate the relationship between protein hydrophobicity and the pyrimidine content of the genes, we plot in Figure 4 the percent pyrimidine in the protein transcript versus the grand average of hydropathicity (GRAVY) of the protein for four species with numerous mitochondrial-encoded soluble and membrane protein genes. A higher GRAVY score indicates a higher hydrophobicity of the protein. There was a strong correlation (*P *< 0.001) between the percent pyrimidine and the hydrophobicity of the encoded protein. This correlation has been shown previously for transcripts of nuclear-encoded proteins [10] and for the second codon position in mitochondrial transcripts from animals and other metazoan mitochondrial genomes that strictly encode membrane proteins [11]. We show that the correlation holds nicely for entire mitochondrial protein transcripts, whether the proteins are soluble or membrane-bound. At high GRAVY scores there is a consistent excursion of membrane proteins from the correlation line. Also, the membrane proteins having low GRAVY scores and low pyrimidine content in the transcripts are likely peripheral membrane proteins. Interestingly, the strong correlation in Figure 4 also holds for *Dictyostelium *where almost all mitochondrial transcripts are purine-rich.

**Figure 5:**
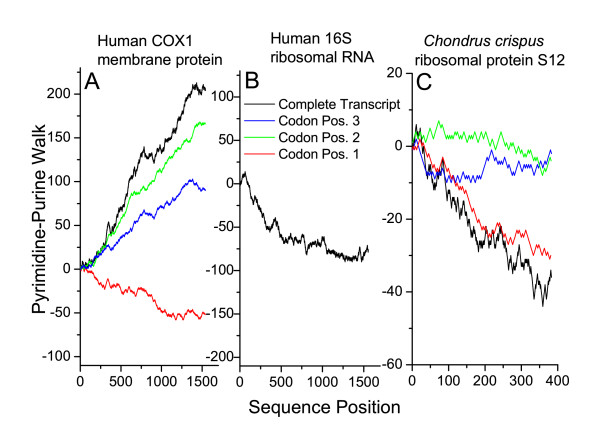
**Pyrimidine-purine codon position walks of select mitochondrial-encoded transcripts**. (A) Membrane protein transcript, human COX1 (C) Soluble protein transcript, *Chondrus crispus *ribosomal protein S12. For each codon position step, the x-axis was incremented by 3 for comparison to the complete transcript. A pyrimidine-purine walk of (B) human 16S ribosomal RNA is also shown for comparison.

It has been noted that the hydrophobicity of a protein is related to the pyrimidine content of position 2 in the codons of the gene [11,12]. If this is the cause of the pattern that we see in the mitochondrial protein genes, then by splitting the DNA walk into three separate walks, one for each codon position, we would expect that the walk using codon position 2 would be responsible for the signal, while the walks for codon positions 1 and 3 might be random. Fig. 5 shows a pyrimidine-purine walk of each codon position of the human COX1 membrane protein transcript and the *Chondrus crispus *S12 soluble ribosomal protein transcript. For comparison, the pyrimidine-purine walk of the human 16S ribosomal RNA is also shown. The base composition of mitochondrial genome sections encoding rRNA and tRNA from other species is given in additional file 1: Table S1. These walks are given as examples to show the uniformity of the signal along the length of the gene. The mitochondrial-encoded transcripts from other species have a similar pattern of pyrimidine-richness in the three codon positions (see Figures 3B and 3C and Table 2). In mtDNA-encoded membrane-protein transcripts, codon position 2 contains the most pyrimidines, as predicted (Figure 3B). However, codon position 3 also contributes slightly to the pyrimidine-rich signal while codon position 1 is often slightly purine-rich. In the soluble ribosomal protein transcript the purine-rich signal is driven mainly by codon position 1, while codon positions 2 and 3 contribute only slightly (see also Figure 3B). The eight known mtDNA-encoded soluble protein transcripts from *Arabidopsis *give a similar purine-rich signal in pyrimidine-purine walks (see additional file 1: Figure S1). Even the signals of the individual codon positions follow the same trends in all eight genes.

**Table 2:** Base composition at each codon position in mtDNA-encoded membrane and soluble protein-coding transcripts. Analysis was performed on transcripts from humans and the species from Table 1 that encode soluble proteins in mtDNA.

	Membrane Proteins						Soluble Proteins					
	%A	%G	%C	%T	%Pu	%AT	%A	%G	%C	%T	%Pu	%AT

*Saccharomyces cerevisiae*												
Codon pos. 1	28.1	26.4	10.3	35.2	54.5	63.3	42.5	20	8.5	29.1	62.4	71.6
Codon pos. 2	21.3	12.6	22	44.1	33.9	65.3	36.4	13.3	15	35.3	49.6	71.6
Codon pos. 3	42.9	5.14	7.49	44.5	48	87.4	42.5	4.57	3.9	49	47.1	91.5
All	30.7	14.7	13.3	41.3	45.5	72	40.5	12.6	9.2	37.8	53.1	78.3
*Arabidopsis thaliana*												
Codon pos. 1	26	27.6	20.5	26	53.6	51.9	33	26	22	19.1	59.1	52.1
Codon pos. 2	21.5	17.7	23.7	37.2	39.2	58.6	29	22	22	27.4	51	56.5
Codon pos. 3	26.5	17.8	17.5	38.2	44.3	64.8	29.8	22.2	22	26.3	51.9	56.1
All	24.7	21	20.5	33.8	45.7	58.4	30.6	23.4	22	24.3	54	54.9
*Porphyra purpurea*												
Codon pos. 1	30.8	23.4	14.7	31.1	54.2	61.9	41.8	21.2	14	23.1	63	64.9
Codon pos. 2	21.5	14.6	20.9	43	36.1	64.4	37.6	14.9	18	29.4	52.5	67.1
Codon pos. 3	34.9	11.4	15.4	38.3	46.3	73.2	39.9	12.2	14	34	52.1	73.9
All	29.1	16.5	17	37.5	45.5	66.5	39.8	16.1	15	28.8	55.9	68.6
*Dictyostelium discoideum*												
Codon pos. 1	35.9	28.3	7.92	27.9	64.2	63.8	45.4	23.6	11	20.4	69	65.8
Codon pos. 2	25.7	15.3	15.9	43.1	41	68.8	39.4	17.7	13	30.1	57.2	69.6
Codon pos. 3	57.8	7.24	4.83	30.2	65	87.9	61.2	10.1	4.1	24.6	71.3	85.8
All	39.8	16.9	9.55	33.7	56.7	73.5	48.7	17.2	9.1	25	65.8	73.7
*Chondrus crispus*												
Codon pos. 1	30.5	20.3	13.8	35.4	50.8	65.9	41.8	16.1	14	28.5	57.9	70.3
Codon pos. 2	21.4	13.5	19.8	45.3	34.9	66.7	35.7	13.9	17	33.6	49.6	69.3
Codon pos. 3	35.9	7.92	7.75	48.4	43.8	84.3	46	6.81	7.2	40	52.8	86
All	29.3	13.9	13.8	43.1	43.2	72.3	41.2	12.3	13	34	53.4	75.2
*Marchantia polymorpha*												
Codon pos. 1	27	28.5	16	28.6	55.5	55.5	35.2	23	19	22.9	58.2	58.1
Codon pos. 2	20.8	16.5	21.1	41.6	37.3	62.4	32.7	19.6	19	28.5	52.3	61.1
Codon pos. 3	28.5	14.6	13.9	43	43.1	71.5	36	15.3	13	35.3	51.3	71.3
All	25.4	19.8	17	37.7	45.3	63.2	34.6	19.3	17	28.9	53.9	63.5
*Metridium senile*												
Codon pos. 1	21.7	18.5	19.8	40	40.2	61.7	30.4	28.1	17	24.6	58.5	54.9
Codon pos. 2	25.9	17.7	16.4	40	43.6	65.9	31.7	20.1	17	30.8	51.8	62.5
Codon pos. 3	26.4	25.1	14.7	33.8	51.5	60.2	39.3	13.4	11	36.6	52.7	75.9
All	24.6	20.4	17	37.9	45.1	62.6	33.8	20.5	15	30.7	54.3	64.4
*Chlamydomonas reinhardtii*												
Codon pos. 1	24.7	30.5	17.5	27.2	55.2	51.9	25.2	24.4	32	18.7	49.6	43.9
Codon pos. 2	16.4	19.3	21.5	42.8	35.8	59.2	34.1	14.4	19	32.8	48.5	66.9
Codon pos. 3	15.5	19.8	26.3	38.4	35.3	53.9	21.7	23.6	24	31.2	45.3	52.8
All	18.9	23.2	21.8	36.1	42.1	55	27	20.8	25	27.6	47.8	54.6
*Homo sapiens*												
Codon pos. 1	29.5	19.6	26.9	24	49.1	53.5						
Codon pos. 2	22.8	11.4	29.9	36	34.2	58.8						
Codon pos. 3	34.3	8.38	39	18.4	42.7	52.7						
All	28.9	13.1	31.9	26.1	42	55						

**Figure 6:**
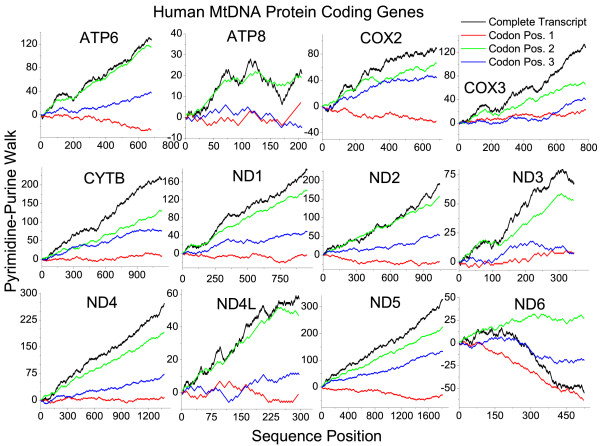
**Pyrimidine-purine codon position walks of human mtDNA-encoded protein transcripts**. The COX1 transcript walk, absent in this figure, is shown in Figure 5A. All 13 genes have similar patterns in the codon positions of the pyrimidine-purine walks.

As an example of the robustness of this signal, pyrimidine-purine walks of each codon position from the other 12 human mtDNA protein-coding genes are shown in Figure 6. From the linearity of the walk of the entire genome in Figure 1, it is clear that the signal strength is almost constant through all protein-coding genes. The pyrimidine-rich signal is driven by codon position 2 in almost all cases, with position 3 contributing modestly and codon position 1 not contributing to an appreciable extent. The conservation of this pattern through the vast majority of the mitochondrial transcripts indicates the strong selective pressure for this signature. Unlike the other transcripts, the ND6 transcript is purine-rich, but it is also the only transcript encoded on the (+) light strand of mtDNA. So there does appear to be a strand-specific selective force present as well, in human mtDNA. However, the ND6 transcript is pyrimidine-rich in other mammals even though it is encoded on the (+) light strand (see Table 1). The percent occurrence of each individual nucleotide species in each codon position in mitochondrial genomes from many eukaryotic species is given in Table 2. It is shown that C more than T in codon positions 1 and 3 drives the pyrimidine-rich signal in human membrane protein transcripts, while T more than C at codon position 2 also contributes. The constancy of this pattern throughout the genes as well as the overall abundance of A over G and C over T can be observed in 2-dimensional walks of the individual codon positions and entire transcripts (see additional file 1: Figure S2). The pyrimidine-rich signal is driven by C over T only in birds, reptiles, and some mammalian species. In all other species examined, T drives the signal from all 3 codon positions.

## Discussion

### Mitochondrial-encoded membrane protein transcripts are pyrimidine-rich

Protein transcripts with an abundance of U (T) in the second codon position encode hydrophobic amino acids [10,13] that tend to form membrane-spanning alpha helices [14] or beta strands [12]. This is likely the most important factor that contributes to the relative pyrimidine-richness of mitochondrial membrane complex transcripts. However it does not explain the entire signal in humans where large quantities of C in the third codon position also play an important role. In fact, the pyrimidine-rich signal in humans is mainly driven by C in the third codon position (Table 2). The signal is also partially driven by the lack of G in the transcripts. Mitochondrial DNA is replicated by a strand asymmetric mechanism [15] that is likely responsible for the unequal strand distribution of G nucleotides [16]. G is the most easily oxidized base, forming 8-hydroxy guanine [17]. A low percentage of G in the vertebrate mitochondrial transcripts has been hypothesized to contribute to mRNA stability in the oxidative environment of the matrix space [18]. However, we must emphasize that the low G abundance in the light strand in vertebrates is not the primary source of the pyrimidine richness in these transcripts, because membrane protein transcripts are also pyrimidine-rich in species where no mitochondrial strand asymmetry in G content is present (see Table 2).

The relative contribution of C versus T, and A versus G throughout the transcripts can be seen in the 2-dimensional walks of the genes using A-G on one axis and C-T on the other (additional file 1: Figure S2). The percentage of C vs. T has been shown to vary greatly in different mammalian lineages [7]. The greater abundance of C over T on the mitochondrial light (+) strand first appears evolutionarily in reptiles and is accompanied by a slightly more G-C rich mitochondrial genome (37 % in *Xenopus *compared to 44 % in humans (additional file 1: Table S1)). The development of GC-rich isochores also first occurred in the nuclear DNA of reptiles and may be one of the factors allowing the evolution of warm-blooded birds and mammals [19]. Based on the data presented here, some of the same selective pressures may be affecting both the nuclear and mitochondrial genomes.

### Mitochondrial-encoded soluble protein transcripts are purine-rich

It has been suggested that purine-loading of transcripts may have evolved to prevent detrimental RNA-RNA interactions [20]. However this hypothesis does not explain the codon-specific pattern of purine-richness in mitochondrial soluble protein-coding transcripts. An A in the second codon position of nuclear-encoded transcripts often encodes relatively hydrophilic amino acids [12]. These amino acids have been shown to be abundant in the aperiodic secondary structure of soluble proteins. However, in the mitochondrial genome, purines (see Figure 3C), specifically A, in the first codon position (not the second) mainly drives the purine-richness of soluble proteins (see Table 2). To the best of our knowledge, purine abundance in the first codon position has not previously been associated with the hydrophilic nature of soluble proteins, even though this signature does occur in the vast majority of nuclear-encoded transcripts [21,22]. One hypothesis that could be tested is that ribosomes translate more efficiently when purines are present at the first codon position. Additionally, increased levels of specific tRNAs in the mitochondrial matrix may select for such a trend. However A in the second codon position also contributes to the signal. A decrease in T nucleotides accompanies the increase in A nucleotides in both positions. The result of this A for T substitution in the first two codon positions is the greater abundance of the hydrophilic amino acids lysine and asparagine (codon AAX) in soluble mitochondrial proteins and the decreased abundance of the hydrophobic amino acids phenylalanine and leucine (codons UUX and CUX). In fact much of the purine-rich signal in the soluble proteins is due to the 3–4 fold increase in positively charged lysine residues in these proteins compared to membrane proteins (data not shown). Mitochondrial ribosomal proteins use these residues to bind the negatively charged phosphate backbone of ribosomal RNA [23,24].

### Mitochondrial ribosomal RNA is purine-rich

The selective pressure that maintains the slight purine richness of mitochondrial ribosomal RNA is not entirely clear [25,26]. It is known that ribosomal RNA interacts with ribosomal protein through hydrophobic interactions of unpaired A residues in the RNA loop regions with hydrophobic protein side chains [27,28]. Purine nucleotides are more hydrophobic than pyrimidine nucleotides [13,29]. Therefore this slight purine abundance in the loop regions may be conserved to facilitate this interaction. Mitochondrial introns are also purine-rich (additional file 1: Figure S3), likely conserving a hydrophobic interaction between splicing proteins and the loop structures in the RNA.

It is difficult to hypothesize how such small magnitutudes of purine and pyrimidine base skew can be conserved over the billion years of mitochondrial evolution. Skewed ribonucleoside triphosphate pools (highest in ATP) [30] may select for a high level of A (purine) in ribosomal RNA and soluble protein transcripts while the need for hydrophobicity in membrane proteins may overcome this pressure, resulting in pyrimidine-rich transcripts. The selective pressure to contain charged hydrophilic amino acids in soluble proteins may also contribute to the maintenance of the purine-rich signal in soluble protein transcripts as well as the abundance of hydrophobic A residues in the loop regions of ribosomal RNA. A better understanding of these mitochondrial selection pressures may be gained in the future by comparing the pyrimidine-purine transcript asymmetries with that of non-coding mtDNA.

## Methods

Mitochondrial gene sequences, amino acid sequences and genomes were downloaded from the NCBI website. Java (JDK 1.50) programs were written to analyze the sequences. The programs or software details are available from the authors upon request. Other websites such as the OGRe database of mitochondrial genomes [31] also allow analysis and graphing of base composition at the codon positions as well. The calculations were performed on a 2.8 GHz desktop computer and typically took less than a few seconds to run. The gene sequences analyzed are the mRNA synonymous sequences as available in PubMed. The mitochondrial genome strand labels (+) and (-) follow PubMed convention.

## Authors' contributions

PB generated the data, constructed Figures 3, 4, 5, 6 and drafted the manuscript. AR wrote the majority of the computer code for sequence analysis, generated the data for and constructed Figures 1 and 2. DC designed and supervised the study.

## Supplementary Material

Additional File 1 Table S1 Base percentages in the entire mitochondrial genome and in just protein-coding or RNA-coding sections. Figure S1 The pyrimidine-purine walk of each codon position of eight soluble mitochondrial-encoded protein transcripts from *Arabidopsis thaliana*. Figure S2 The 2-dimensional A-G and T-C walks of human mtDNA-encoded transcripts. Figure S3 Pyrimidine-purine walks of unspliced mitochondrial-encoded transcripts from *Arabidopsis thaliana *and *Marchantia polymorpha*. The introns in these transcripts do not encode known proteins. Click here for file
