# Supplementary material for: Mitochondrial-encoded membrane protein transcripts are pyrimidine-rich while soluble protein transcripts and ribosomal RNA are purine-rich
Source: BMC Genomics. 2005 Sep 26;6:136. doi: 10.1186/1471-2164-6-136 (PMC1262711; doi:10.1186/1471-2164-6-136)
Supplement: Additional File 1 — Table S1 Base percentages in the entire mitochondrial genome and in just protein-coding or RNA-coding sections. Figure S1 The pyrimidine-purine walk of each codon position of eight soluble mitochondrial-encoded protein transcripts from Arabidopsis thaliana. Figure S2 The 2-dimensional A-G and T-C walks of human mtDNA-encoded transcripts. Figure S3 Pyrimidine-purine walks of unspliced mitochondrial-encoded transcripts from Arabidopsis thaliana and Marchantia polymorpha. The introns in these transcripts do not encode known proteins. [file 1471-2164-6-136-S1.pdf]

Addition file 1

for

Mitochondrial-encoded membrane protein transcripts are pyrimidine-rich while soluble protein transcripts and ribosomal RNA are purine-rich

By Patrick C. Bradshaw, Anand Rathi, and David C. Samuels

**Species**	**Genome** %Pur	%AT	**RNA-Coding** %Pur	%Pyrim	Length	%A	%G	%C	%T	%AT
Homo sapiens (Human)	44	55.5	51.9	48.1	4024	33.3	18.7	23.1	25	58.3
Mus musculus (Mouse)	45.3	62.9	53.5	46.5	4038	36.7	16.9	18.2	28.3	65
Gallus gallus (Chicken)	43.8	54	51.2	48.8	4200	31.8	19.4	25.8	23	54.8
Alligator mississippiensis (Alligator)	44.8	57	52.7	47.3	4158	33	19.6	24.3	23	56.1
Xenopus laevis (African clawed frog)	46.6	63	52.8	47.2	3988	32.8	20	21.5	25.6	58.5
Danio rerio (Zebrafish)	46	60.7	54.6	45.4	4197	33.3	21.3	22	23.4	56.7
Drosophila melanogaster (Fruit fly)	49.3	82.2	51.9	48.1	3568	39.7	12.2	7.82	40.3	80
Strongylocentrotus purpur. (Sea urchin)	45.1	58.7	54.1	45.9	3953	33.5	20.6	19.3	26.7	60.2
Caenorhabditis elegans (Nematode)	46.3	76.2	51.4	48.6	2889	35.8	15.6	8.69	39.9	75.7
Metridium senile (Sea anemone)	48.2	61.9	56.9	43.1	3412	32.1	24.8	17.4	25.6	57.7
Saccharomyces cerevi. (Baker's yeast)	51.3	82.9	54	46	8410	40.4	13.7	9.82	36.1	76.5
Dictyostelium discoideum (Slime mold)	60.5	72.6	57.9	42.1	6444	36	21.8	13.8	28.3	64.3
Chondrus Crispus (Irish moss)	52.2	72.1	54.6	45.4	6280	36.2	18.3	13.9	31.6	67.8
Porphyra purpurea (Red algae)	53	66.5	55.4	44.6	10769	35	20.4	16.1	28.5	63.5
Marchantia polymorpha (Liverwort)	49.9	57.6	54.5	45.5	11202	28.5	26	21.6	23.8	52.3
Arabidopsis thaliana (Thale cress)	50.2	55.2	50.9	49.1	51413	26.8	24	22.7	26.4	53.3
Chlamydomonas reinhardtii (Grn algae)	50.5	54.8	53.6	46.4	3837	29.5	24.1	21.3	25.1	54.7
Pedinomonas minor (Green algae)	44.1	77.8	51.1	48.9	3969	36.4	14.7	10.3	38.6	75
Physarum polycephalum (Slime mold)	51.3	74.1								
Schizosacc. Pombe (Fission yeast)	49.5	69.9	52.7	47.3	6107	33.5	19.3	14.8	32.5	66
Reclinomonas Americana (Protozoan)	51.5	73.9	55.1	44.9	7246	30.9	24.1	17.4	27.6	58.5
Plasmodium falciparum (Malaria)	48.3	68.4								
Polysphondylium pallidum (Slime mold)	55.9	77.2	57.4	42.6	6267	37.3	20.2	12.4	30.1	67.4

	Soluble Proteins	**Protein-Coding** %Pur	%Pyrim	Length	%A	%G	%C	%T	%AT
Homo sapiens (Human)	no	42	58	11389	28.9	13.1	31.9	26.1	55
Mus musculus (Mouse)	no	44.8	55.2	11403	32.6	12.2	25.1	30.1	62.7
Gallus gallus (Chicken)	no	41.6	58.4	11396	28.3	13.4	33.6	24.8	53.1
Alligator mississippiensis (Alligator)	no	42.6	57.4	11398	29.1	13.4	30	27.4	56.5
Xenopus laevis (African clawed frog)	no	44	56	11375	30.6	13.4	23.8	32.2	62.8
Danio rerio (Zebrafish)	no	45.3	54.7	11409	29.8	15.5	23.8	30.9	60.7
Drosophila melanogaster (Fruit fly)	no	44.4	55.6	11181	32.4	11.9	10.8	44.8	77.2
Strongylocentrotus purpur. (Sea urchin)	no	44.1	55.9	11507	26.1	18	23.5	32.4	58.5
Caenorhabditis elegans (Nematode)	no	44.8	55.2	10299	29.6	15.3	9.22	45.9	75.5
Metridium senile (Sea anemone)	1	45.6	54.4	12600	25.1	20.4	16.9	37.6	62.7
Saccharomyces cerevi. (Baker's yeast)	yes	51.4	48.6	24429	38.5	12.8	9.94	38.7	77.2
Dictyostelium discoideum (Slime mold)	yes	60.8	39.2	40053	44.5	16.2	9.73	29.5	74
Chondrus Crispus (Irish moss)	yes	44.5	55.5	18390	30.9	13.5	13.6	41.9	72.9
Porphyra purpurea (Red algae)	yes	49.4	50.6	25917	32.8	16.6	16.6	34	66.9
Marchantia polymorpha (Liverwort)	yes	50.5	49.5	68999	29.2	21.3	19.2	30.3	59.4
Arabidopsis thaliana (Thale cress)	yes	48.3	51.7	68940	26.3	22	22.2	29.6	55.9
Chlamydomonas reinhardtii (Grn algae)	1	42.8	57.2	9261	19.9	22.9	22.1	35.1	55
Pedinomonas minor (Green algae)	no	39.5	60.5	10398	26.3	13.2	9.71	50.8	77.1
Physarum polycephalum (Slime mold)	yes	52	48	21039	40.3	11.7	12.2	35.7	76.1
Schizosacc. Pombe (Fission yeast)	yes	48.4	51.6	9907	33.5	14.9	14.4	37.2	70.7
Reclinomonas Americana (Protozoan)	yes	51.6	48.4	55920	37.5	14.1	11.2	37.1	74.6
Plasmodium falciparum (Malaria)	no	45.2	54.8	3510	31.1	14	13.5	41.3	72.4
Polysphondylium pallidum (Slime mold)	yes	55.4	44.6	38490	41.7	13.6	7.97	36.7	78.4

Table S1. Percent purine of the entire mitochondrial genome (+ strand) of selected species and individual base composition in just the protein-coding or just the RNA-coding (both tRNA and rRNA) sections.

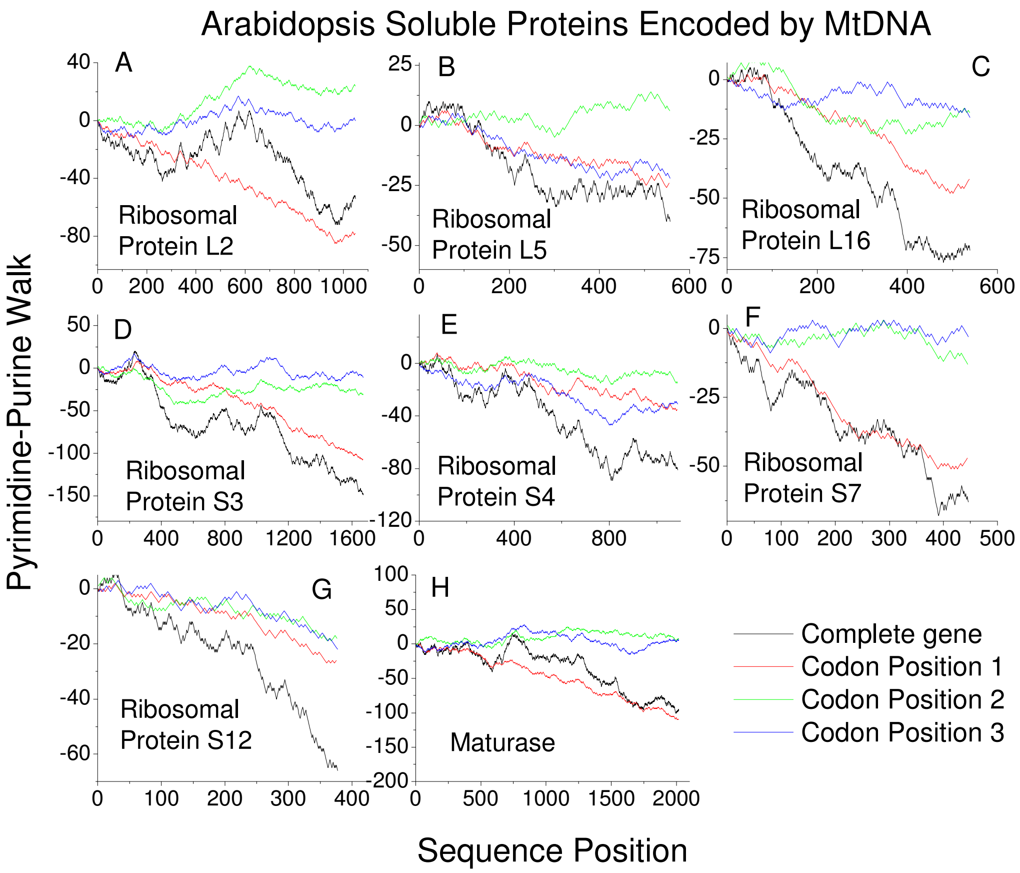

Figure S1 The pyrimidine-purine walk of each codon position of eight soluble mitochondrial-encoded protein transcripts from *Arabidopsis thaliana*.

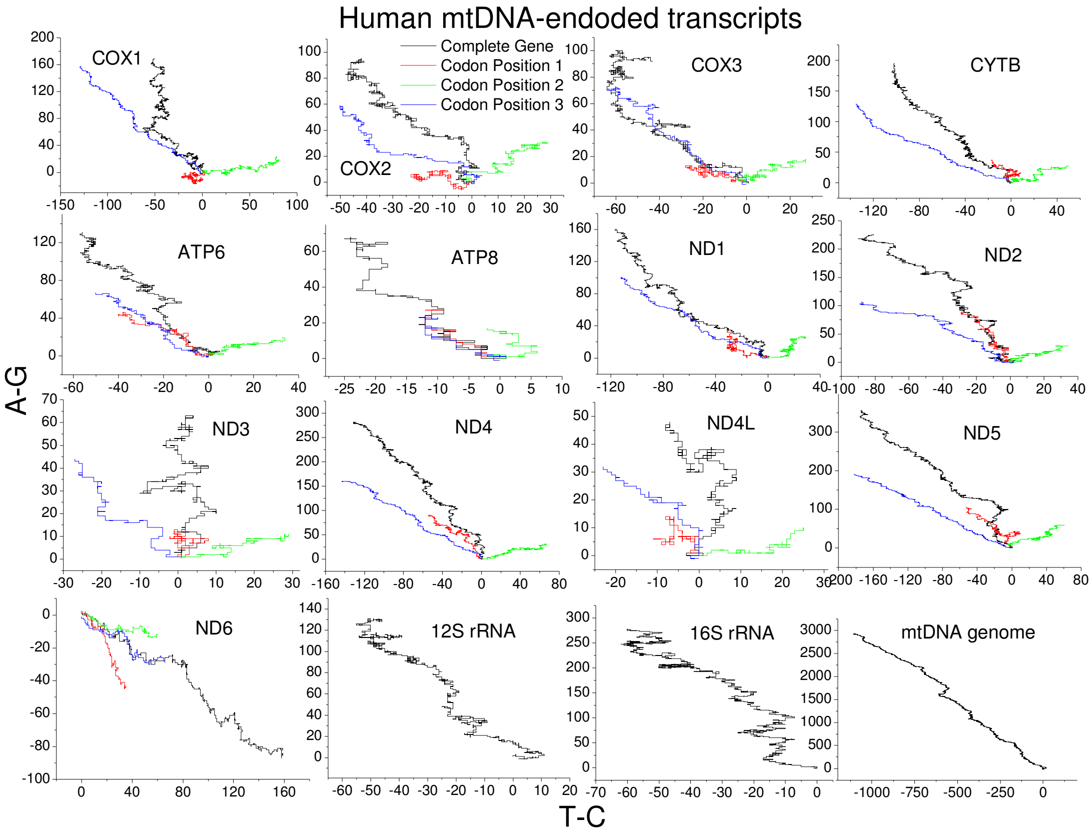

Figure S2 The 2-dimensional A-G and T-C walks of human mtDNA-encoded transcripts.

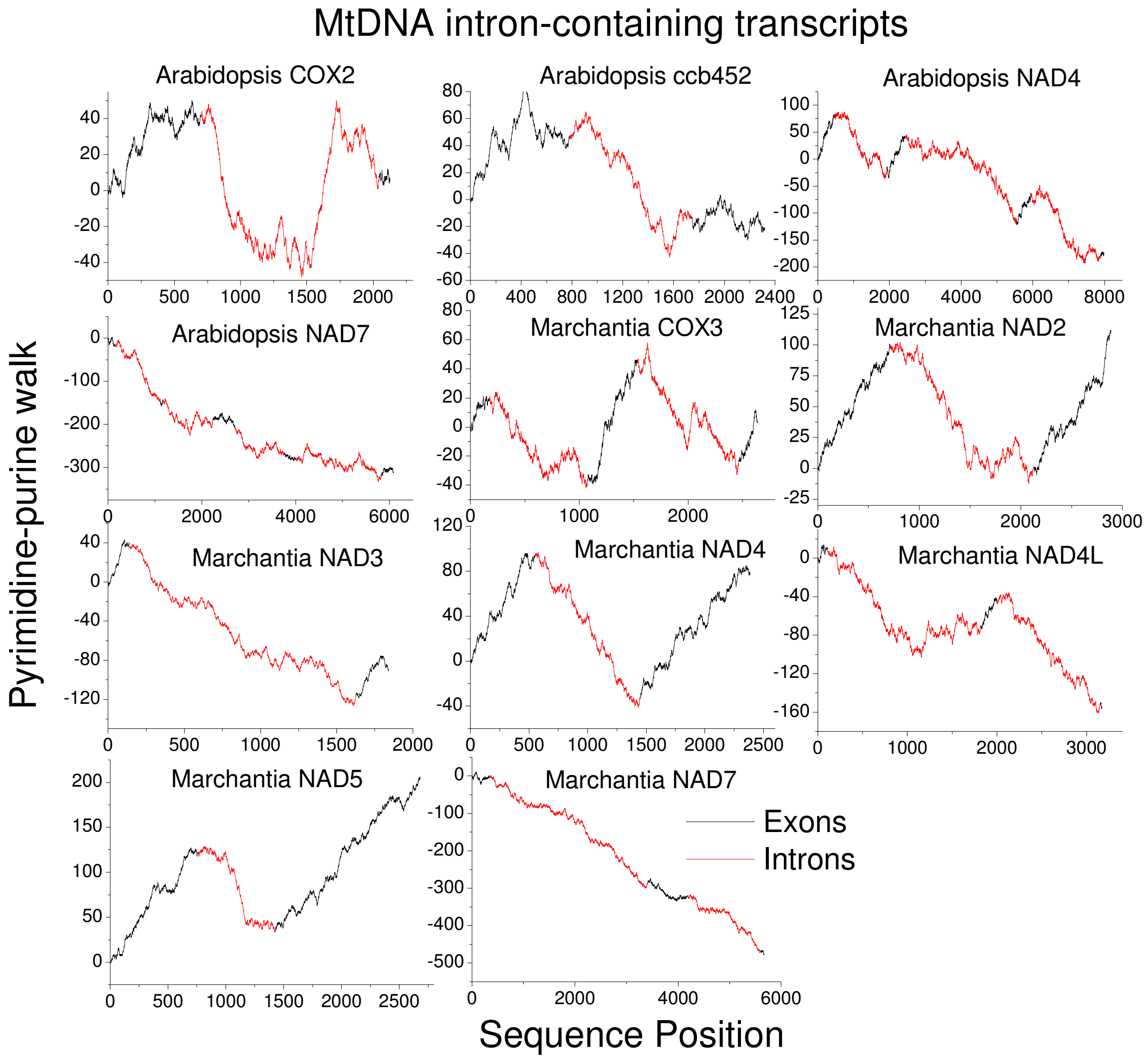

Figure S3 Pyrimidine-purine walks of unspliced mitochondrial-encoded transcripts from *Arabidopsis thaliana* and *Marchantia polymorpha*. The introns in these transcripts do not encode known proteins.
